# Supplementary material for: Hepatic stellate cell stearoyl co-A desaturase activates leukotriene B4 receptor 2 - β-catenin cascade to promote liver tumorigenesis
Source: Nat Commun. 2023 May 8;14:2651. doi: 10.1038/s41467-023-38406-8 (PMC10167314; doi:10.1038/s41467-023-38406-8)
Supplement: Supplementary file 1 — Supplementary Information [file 41467_2023_38406_MOESM1_ESM.pdf]

Supplementary Information

# Hepatic stellate cell stearoyl co-A desaturase activates leukotriene B4 receptor 2-β-catenin cascade to promote liver tumorigenesis

Sonal Sinha, Satoka Aizawa, Yasuhiro Nakano, Alexander Rialdi, Hye Yeon Choi, Rajan Shrestha[1] Stephanie Q Pan, Yibu Chen, Meng Li, Audrey Kapelanski-Lamoureux, Gregory Yochum, Linda Sher, Satdarshan Paul Monga[8] Anthoula Lazaris, Keigo Machida, Michael Karin, Ernesto Guccione, Hidekazu Tsukamoto

Contents

1. Supplementary Table 1: List of antibodies used -page 2
2. Supplementary Table 2: List of qPCR primers and qRNAs used -page 3
3. Supplementary Table 3: Information on HCC Patients -page 4
4. Supplementary Figure 1-4 -page 5-8

**Supplementary Table 1: List of Antibodies Used**

| Antibody | Vendor | Catalog | Condition | Dilution |
|---|---|---|---|---|
| Mouse-Beta-actin | Thermo Fisher | sc-47778 | IB | 1:1000 |
| Rabbit-CTNNB1 | Abcam | ab 32572 | IB | 1:5000 |
| Rabbit-p-ERK1/2 | Cell Signaling | 9101 s | IB | 1:1000 |
| mouse-ERK | Cell Signaling | 9107 s | IB | 1:1000 |
| Mouse-GAPDH | Thermo Fisher | SC-32233 | IB | 1:1000 |
| Rabbit-p-(S9) GSK3$\beta$ | Cell Signaling | 5558 s | IB | 1:1000 |
| Mouse-GSK3$\beta$ | Cell Signaling | 12456 | IB | 1:1000 |
| Rabbit-HuR | Novus MBL | En004p | IB | 1:1000 |
| Rabbit-LTB4R2 | Abcam | ab 8460 | IB | 1:500 |
| Mouse-Lamin-B2 | Cell Signaling | 13823 s | IB | 1:500 |
| Rabbit-LRP-6 | Abcam | ab 66156 | IB | 1:1000 |
| Rat-NaKATPase | Cell Signaling | 3010 s | IB | 1:1000 |
| Rabbit-p-(S127)YAP | Cell Signaling | 13008 s | IB | 1:1000 |
| Rabbit-YAP | Cell Signaling | 4912 s | IB, IF | 1:1000, 1:100 |
| Rabbit-YAP | Cell Signaling | 14074 | IF | 1:400 |
| Mouse-α-SMA | Sigma-Aldrich | A2547 | IF | 1:500 |
| Goat-HNF4α | Santa Cruz | Sc-6556 | IF | 1:200 |
| Mouse-SOX9 | Millipore sigma | AB5603 | IF | 1:500 |
| Rabbit- CYP1B1 | Boster Biological Technology | PB9546 | IHC | 1:1000 |
| Rabbit -LTB4R2 | MyBioSource | MBS243185 | IHC | 1:200 |
| Mouse-AFP | Invitrogen | PA5-21004 | IF | 1:200 |
| Goat-GFP | Rockland | P/A-600-101-215 | IF | 1:400 |
| Rabbit-TAZ | Cell Signaling | 70148s | IB | 1:1000 |
| | | | | |

**Supplementary Table 2: List of qPCR Primers and gRNAs:**

| Gene | Sequence | |
|---|---|---|
| h-YAP | FW: CCTTCTTCAAGCCGCCGGAG; | REV: CAGTGTCCCAGGAGAAACAGC |
| h-SCD1 | FW: GCAGGACGATATCTCTAGCT | REV: GTCTCCACCTTATCTCCTCCATT |
| h-LTB4R2 | FW: TACCACGCAGTCAACCTTCTG | REV: GCGGTGAAGACGTAGAGCAC |
| h-CTGF | FW: CAGCATGGACGTTCGTCTG | REV: AACCACGGTTTGGTCCTTGG |
| h-CYR61 | FW: CTCGCTTAGTCGTCACCC | REV: CGCCGAAGTTGCATTCCAG |
| h-LRG5 | FW: GAGTTACGTCTTGCGGGAAAC | REV: TGGGTACGTGTCTTAGCTGATTA |
| h-PTGS1 | FW: TTGACCGCTACCAGTGTGAC | REV: GGAAGTGGGTGAAAGAGGGG |
| h-PTGS2 | FW: CCCCAGGGCTCAAACATGAT | REV: ACCGTAGATGCTCAGGGACT |
| h-TBXAS1 | FW: CCGAGACGAACTGAATGGCT | REV: GGCATCCAGGACCATTTGGA |
| h-CYP1A1 | FW: TCGGCCACGGAGTTTCTTC | REV: GGTCAGCATGTGCCCAATCA |
| h-CYP1B1 | FW: TGAGTGCCGTGTGTTTCGG | REV: GTTGCTGAAGTTGCGGTTGAG |
| h-ALOX15 | FW: CAGCGTGGAAAACAGTG | REV: TCTAGGGAGGGTGGGACATG |
| h-ALOX12 | FW: CTTCTCCGGGTCGTACAACC | REV: CCAAGTCCTCTGCAACGTCA |
| h-BRIC | FW: AGCATTCGTCCGGTTGCGCT | REV: TCGATGGCACGGCGACTTT |
| h-CCND1 | FW: GGTGGCCGCAGTGCAA | REV: GAAGCGTGTGAGGCGGTAGTA |
| h-BCL2L1 | FW: TGGACAATGGACTGGTTGAG | REV: GGGAAAGCTTGTAGGAGAGAAA |
| h-36B4 | FW: TCTTGGGACAAATTGGACATGG | REV: GTGAGCCACTTGGTGTTGGA |
| m-36B4 | FW: AGATTCGGGATATGCTGTTGGC | REV: TCGGGTCCTAGACCAGTGTTC |
| m-Cnnd1 | FW: TGCAAATGGAACTGCTTCTG | REV: TGGAAAGAAAGTGCGTTGTG |
| m-Yap | FW: ATGACAACCAATAGTTCCGATCC | REV: CAGGGTGCTTTGGCTGATG |
| m-Ctgf | FW: AGAACTGTGTACGGAGCGTG | REV: GTGCACCATCTTTGGCAGTG |
| m-Cyr61 | FW: AGAGGCTTCCTGTCTCTTTGGC | REV: CCAAGACGTGGTCTGAACGA |
| m-Bric5 | FW: TGCAAAGGAGACCAACAACA | REV: GGCATGTCACTCAGGTCCAA |
| m-Ltb4r2 | FW: ACAGCCTTGGCTTTCTTCAG | REV: TGCCCCATTACTTTCAGCTT |
| m-Scd1 | FW: TACACTCTGGTGCTCAACGC | REV: AGGATATTCTCCCGGGATTG |
| m-Scd2 | FW: GCTCTCGGGAGAACATCTTG | REV: CAGCCCTGGACACTCTCTCTTC |
| m-Glul | FW: TGAACAAAGGCATCAAGCAAATG | REV: CAGTCCAGGGTACGGGTCTT |
| m-Col1α1 | FW: CACCCTCAAGAGCCTGAGTC | REV: GTTCGGGCTGATGTACCAGT |
| m-Wwtr1 | FW: CATGGCGGAAAAAGATCCTCC | REV:GTCGGTCACGTCATAGGACTG |
| h-CYP1B1 gRNA 1 | gRNA TGCGCCCGAACTCTTCGTTG | |
| h-CYP1B1 gRNA 2 | gRNA ACTGATCGGAAACGCGGCGG | |
| h-CYP1B1 gRNA control | gRNA AAAGTCGCCCTCCGCGCTCG | |

Supplementary Table 3: Summary of HCC patients whose tissues were analyzed.

| Tissue Type | Diagnosis | Etiology | Sex | Age | Race | Ethnicity |
|---|---|---|---|---|---|---|
| Snap-frozen tissues | HCC/cirrhosis | 3 ALD<br>1 NASH<br>2 HCV | 4 males<br>2 females | $64 \pm 8.3$ | White | 1 Hispanic<br>5 non-Hispanic |
| Paraffin sections | HCC/cirrhosis | 1 ALD<br>1 NASH<br>1 HCV<br>1 HBV | 3 males<br>1 female | $72 \pm 7.5$ | Asian<br>White | 1 Asian<br>3 non-Asian |

# Supplementary Figure 1

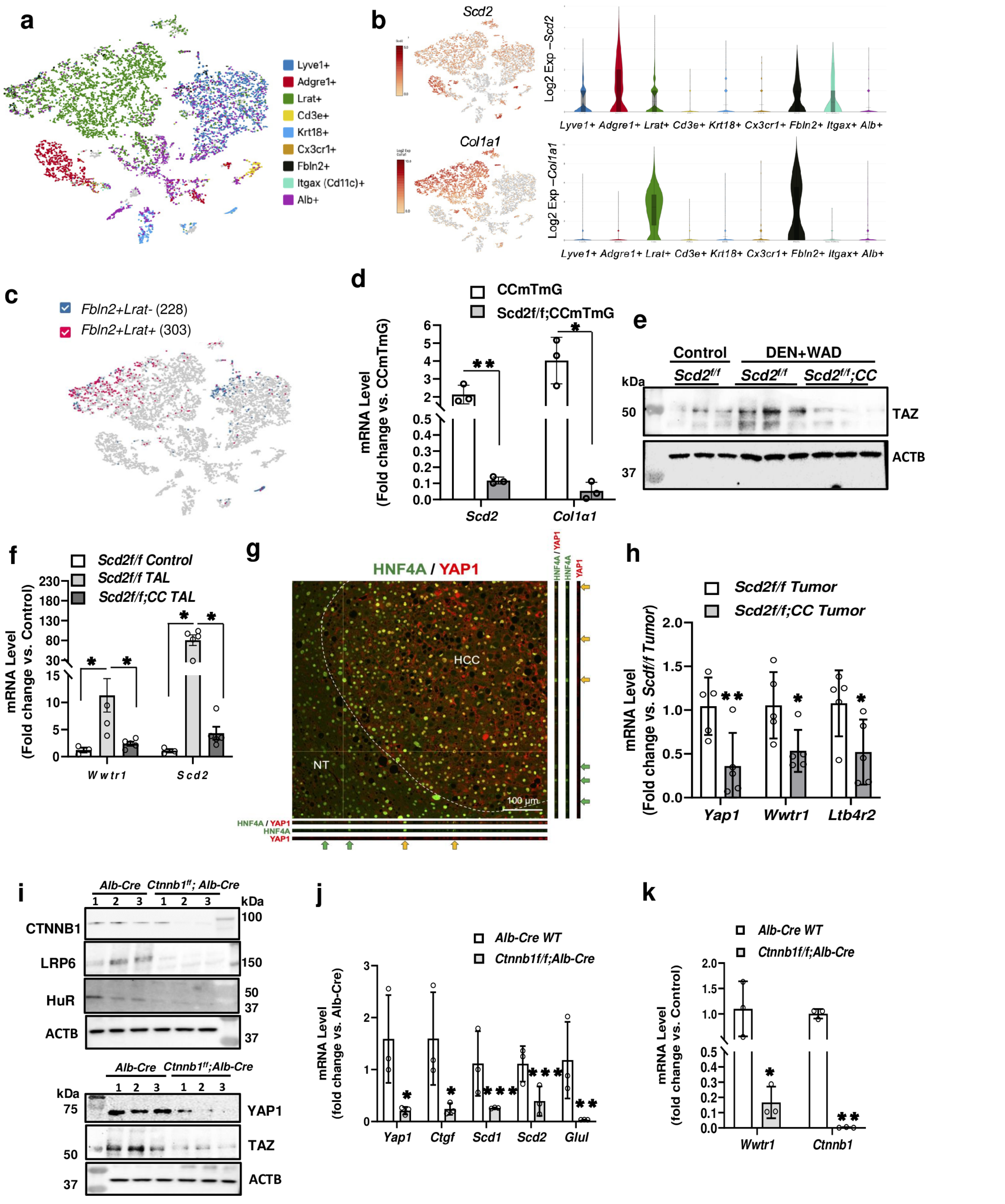


**Supplementary Figure 1. Global YAP1/TAZ repression by SCD2 deficiency in aHSC. a** A t-SNE plot depicting TME cell type clusters in the DEN+WAD mouse liver identified by scRNA-seq using the specific cell type marker genes. **b** *Scd2* and *Col1a1* expressions by the cell type clusters as shown by t-SNE plot and violin plots (Violin plot parameters and cell numbers for different cell types analyzed are provided in Supplementary Data 3 in Supplementary File). **c** A t-SNE plot showing the distribution of *Fbln2+Lrat-* vs. *Fbln2+Lrat+* cells. Numbers in the parenthesis are cell numbers. **d** Effective *Scd2* and *Col1a1* mRNA reductions in HSC from *Scd2*$^{f/f}$*;CC;mTmG* vs. *CC;mTmG* mice. *p<0.05, **p<0.01 by two-sided t-test. Data presented are means±SEM (n=3 pairs). **e** TAZ IB analysis showing its upregulation in *Scd2*$^{f/f}$ *TAL* is blocked in *Scd2*$^{f/f}$*;CC* (n=3 mouse samples each). **f** *Wwtr1* and *Scd2 mRNA* in *Scd2*$^{f/f}$*;CC* vs. *Scd2*$^{f/f}$ TAL vs. normal *Scd2*$^{f/f}$ liver (Control). *p<0.05 by two-sided t-test. Data presented are means±SEM (n=3-5). **g** Representative 3D confocal imaging of HN4A and YAP1 IF staining of *Scd2*$^{f/f}$ TAL section from 4 samples analyzed. **h** *Yap1*, *Wwtr1*, and *Ltb4r2* mRNA levels in *Scd2*$^{f/f}$*;CC* vs. *Scd2*$^{f/f}$ tumors. *p<0.05. Data presented are means±SEM (n=5). *p<0.05, **p<0.01 by two-sided t-test. **i** IB analysis of CTNNB1, HuR, LRP6, YAP1, and TAZ along with the house keeping ACTB in *AlbCre* vs. *Ctnnb1f/f;AlbCre* mouse livers. (n=3 each). **j** CTNNB1 and YAP1-target gene mRNA levels in *AlbCre* vs. *Ctnnb1f/f;AlbCre* mouse livers. *p<0.05, **p<0.01, ***p<0.005 vs. Alb-Cre by two-sided t-test. Data presented are means±SEM (n=3 each). **k** *Wwtr1* and *Ctnnb1* mRNA levels in *AlbCre* vs. *Ctnnb1f/f;AlbCre* mouse livers. *p<0.05, **p<0.01 vs. Alb-Cre by two-sided t-test. Data presented are means±SEM (n=3 each). All relevant figures, source data and exact p values are provided in the Source Data file.

# Supplementary Figure 2

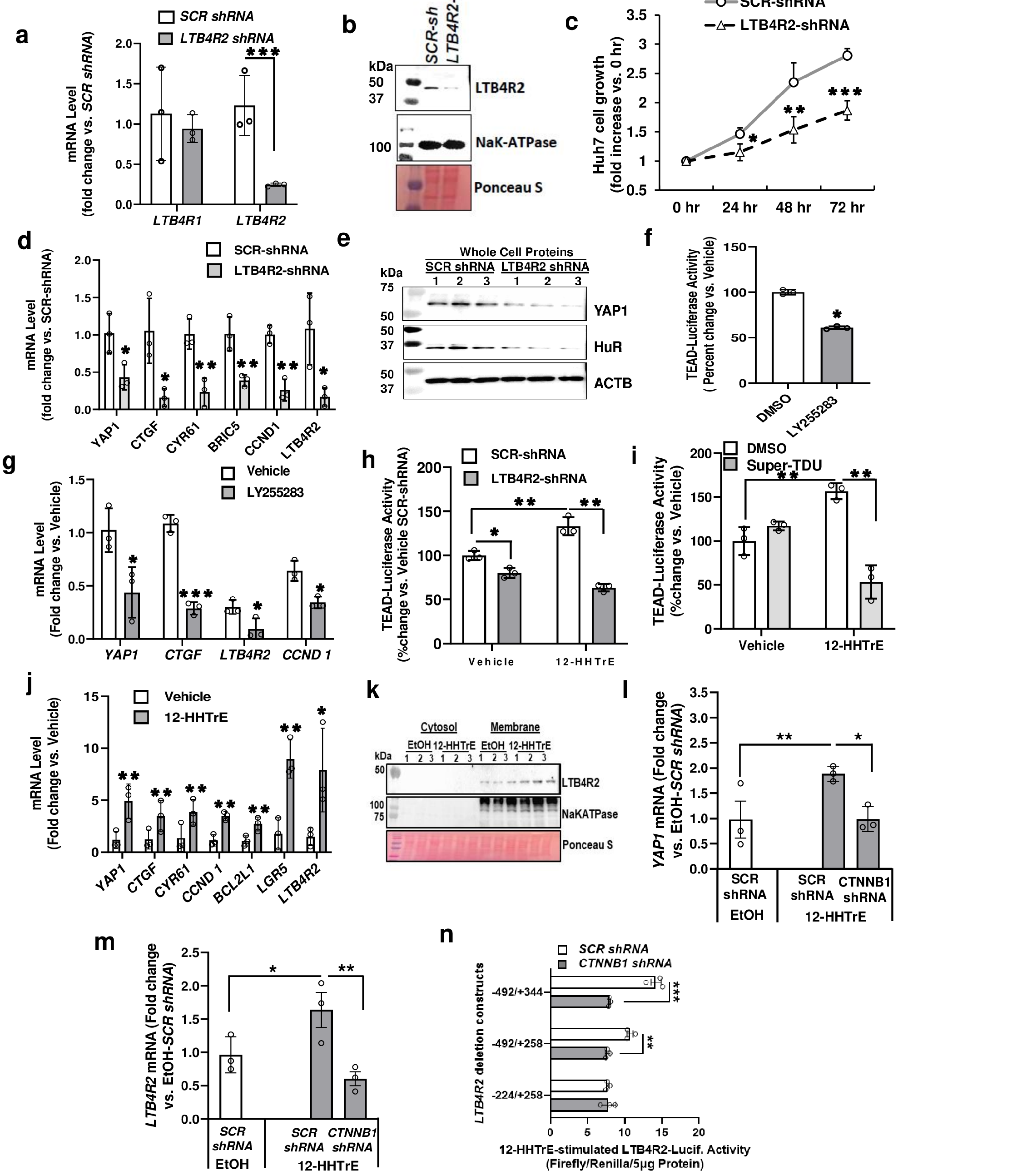

**Supplementary Figure 2. 12-HHTRE-LTB4R2 tumor promoter pathway. a** *LTB4R1* and *LTB4R2* qPCR data for Huh7 cells with adenoviral expression of *LTB4R2-shRNA* vs. *SCR-shRNA* at MOI=15 after 72 hr post-infection. ***p<0.005 vs. *SCD-shRNA* by two-sided t-test. Data presented are means±SEM (n=3 experiments). **b** IB analysis of LTB4R2 in Huh7 cells with *LTB4R2-shRNA* vs. *SCR-shRNA*. (a represented image from 3 experiments). **c** Growth curve for Huh7 cells with *LTB4R2-shRNA* vs. *SCR-shRNA*. *p<0.05, **p<0.01, ***p<0.005 vs. *SCR-shRNA* by two-sided t-test. Data presented are means±SEM (n=3 experiments). **d** mRNA expression of *YAP1* and its target genes (*CTGF, CYR61* and *BRIC5*), CTNNB1-target gene *CCND1*, and *LTB4R2* in *LTB4R2-shRNA* vs. *SCR-shRNA* Huh7 cells. *p<0.05, **p<0.01 vs. *SCR-shRNA* by two-sided t-test. Data shown are means±SEM (n=3 experiments). **e** IB analysis for YAP1 and HuR of whole cell proteins from Huh7 cells with *LTB4R2-shRNA* vs. *SCR-shRNA*. (n=3 replicates). **f** TEAD-luciferase activity in Huh7 cells treated for 24 hr with the receptor antagonist LY255283 (10μM) vs. DMSO (vehicle). *p<0.05 by two-sided t-test. Data presented are means±SEM (n=3 experiments). **g** mRNA levels of *YAP1, CTGF, LTB4R2,* and *CCND1* in Huh7 cells treated with LY255283 (10μM) vs. DMSO. *p<0.05, ***p<0.005 vs. vehicle by two-sided t-test. Data presented are means±SEM (n=3 experiments). **h** TEAD-luciferase activity in Huh7 cells with *LTB4R2-shRNA* vs. *SCR-shRNA* which were treated for 24 hr with 12-HHTrE (50nM) or vehicle (ethanol). *p<0.05, **p<0.01 by two-sided t-test. Data presented are means±SEM (n=3 experiments). **i** TEAD-luciferase activity in Huh7 cells treated with 12-HHTrE (50nM) or vehicle (EtOH) in the presence of Super-TDU (300μM) or DMSO. **p<0.01 by two-sided t-test. Data presented are means±SEM (n=3 experiments). **j** mRNA expression of *YAP1* and its target genes (*CTGF, CYR61*), CTNNB1-target genes (*CCDN1, LRG5*), *BCL2L1* regulated by both YAP1 and CTNNB1 and *LTB4R2* in Huh7 cells treated with 12HHTrE vs. vehicle (EtOH). *p<0.05, **p<0.01 vs. vehicle by two-sided t-test. Data shown are means±SEM (n=3 experiments). **k** IB analysis of LTB4R2 with membrane proteins from Huh7 cells treated with vehicle (-) or 50nM 12-HHTrE (+). (n= 3 experiments). **l** *YAP1* qPCR data in Huh7 cells infected with adenovirus expressing *CTNNB1-shRNA* vs. *SCR-shRNA* (MOI=10) which were subsequently treated with 12-HHTrE vs. EtOH. **p<0.01, *p<0.05 by two-sided t-test. Data presented are means±SEM (n=3 experiments). **m** *LTB4R2* mRNA in the same cell preparations as above. *p<0.05, **p<0.01 by two-sided t-test. Data presented are means±SEM. **n** 12-HHTrE-stimulated LTB4R2-Luciferase activity in Huh7 cells transduced with *SCR-shRNA* vs. *CTNNB1-shRNA.* **p<0.01, ***p<0.005 by two-sided t-test. Data are means±SEM (n=3 experiments). All relevant figures, source data and exact p values are provided in the Source Data file.

# Supplementary Figure 3

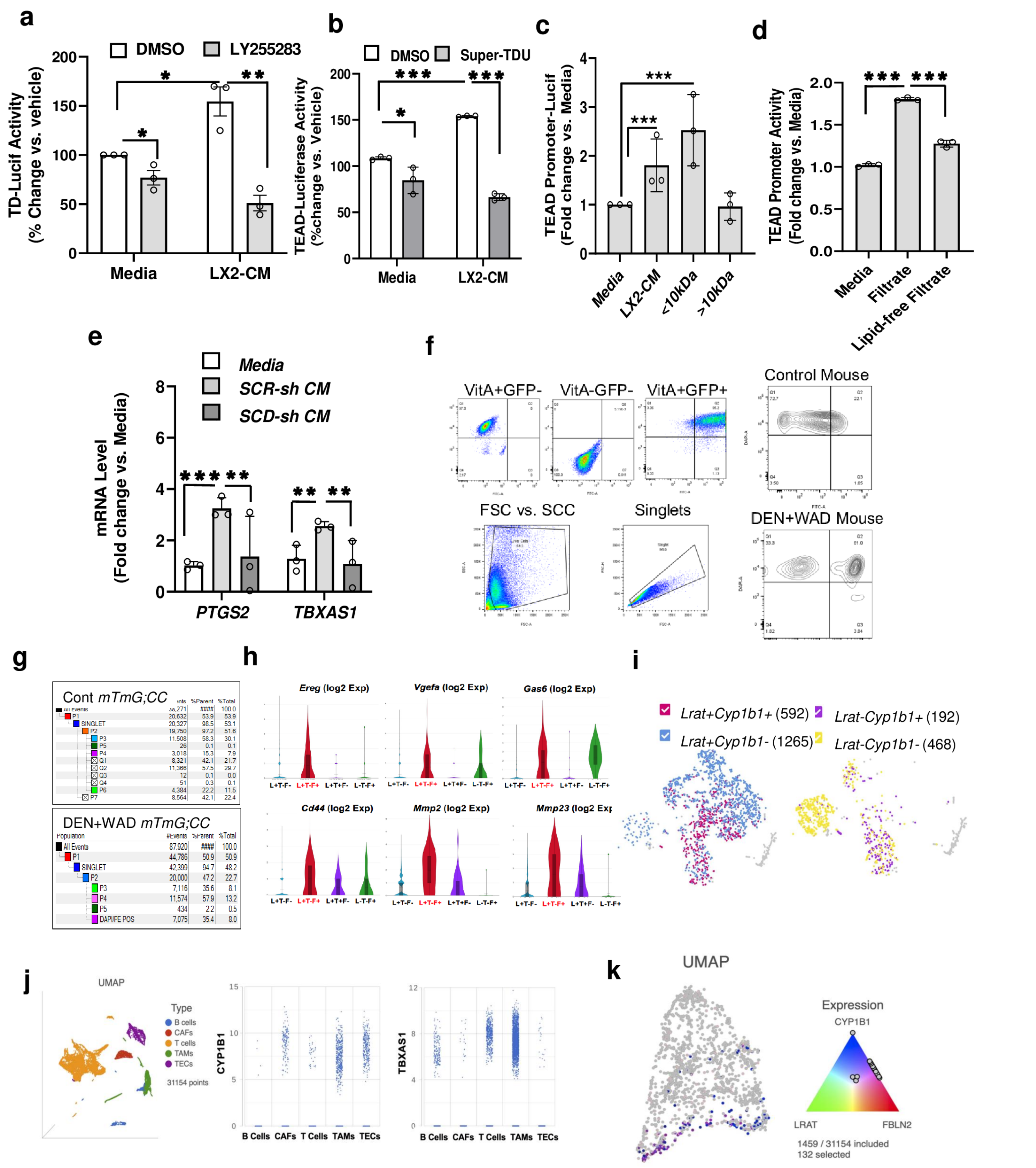


**Supplementary Figure 3. Paracrine aHSC activation of LTB4R2 in HCC cells.** **a** TEAD-luciferase activity in Huh7 cells treated with LX2-CM in the presence of the LTB4R2 receptor antagonist LY255283 (10μM) vs. DMSO. *p<0.05, ***p<0.005 by two-sided t-test. Data presented are means+SEM (n=3 experiments). **b** TEAD-luciferase activity in Huh7 cells treated with LX2-CM in the presence of Super-TDU (300μM) vs. DMSO. *p<0.05, **p<0.01 by two-sided t-test. Data presented are means+SEM (n=3 experiments). **c** TEAD-luciferase activity in Huh7 cells treated with media (no cell), LX2-CM with molecular cut-off of <10 kDa (filtrate) and LX2-CM with molecular cut-off of >10 kDa (retentate). **p<0.01, ***p<0.005 by one-way posthoc ANOVA test. Data presented are means+SEM (n=3 separate experiments). **d** Lipid removal reduces LX2-CM induction of TEA D-luciferase activity. **p<0.05, ***p<0.01 by one-way posthoc ANOVA test. Data presented are means+SEM (n=3 separate experiments). **e** qPCR data for *PTGS2* and *TBXAS1* in Huh7 cells treated with CM collected from LX2 cells with *SCR-shRNA* (SCR-sh CM) vs. *SCD-shRNA* (SCD-sh CM) compared to media control (Media). *p<0.05, **p<0.01 by one-way posthoc ANOVA test. Data presented are means+SEM (n=3 separate experiments). **f** FACS gating strategy. Freshly isolated C57Bl/6j mouse primary HSCs were used as the positive control for VitA+ (DAPI) and negative control for Col1a1GFP (FITC) (VitA+GFP-); the rat myofibroblast cell line BSC cells used as the negative control for both VitA and GFP (VitA-GFP-); and culture-activated HSCs from Col1a1-GFP mice used as the VitA+GFP+ control. Non-parenchymal liver cells were first subjected to forward vs. side scatter (FSC vs. SCC) gating to collect cells of interest which were then subjected to singlet gating for isolation of single cells. The cells were then gated for VitA (DAPI) and GFP (FITC) as set above for isolation of VitA+Col1a1GFP+ activated HSCs (aHSCs) and VitA-Col1a1GFP+ cells from mTmG;CC mice subjected to regular chow feeding without DEN injection (Control) vs. the DEN+WAD tumorigenesis regimen (DEN+WAD). **g** Numbers of cells separated by FACS for VitA+GFP- (P3), VitA+GFP+ (P4), and VitA-GFP+ (P5) populations from mTmG;CC control vs. DEN+WAD mice. **h** Violin plots of tumor promoter genes among VitA-GFP+ subpopulations based on *Lrat*, *Thy1* and *Fbln2* expression (Cell numbers and violin plot parameters of subpopulations are provided in Supplementary Data 3 in Supplementary File). **i** t-SNE plots of VitA-GFP+ cells clustered based on *Lrat* and *Cyp1b1* expression. **j** UMAP of human HCC TME cells from NCI's Single-cell Atlas in Liver Cancer Data (scATlasLC) (left) and CYP1B1 and TBXA1S expression by different cell types (right). **k** CAF co-expressing *CYP1B1* and *FBLN2* via re-analysis of scATlasLC data. All relevant figures, source data and exact p values are provided in the Source Data file.

# Supplementary Figure 4

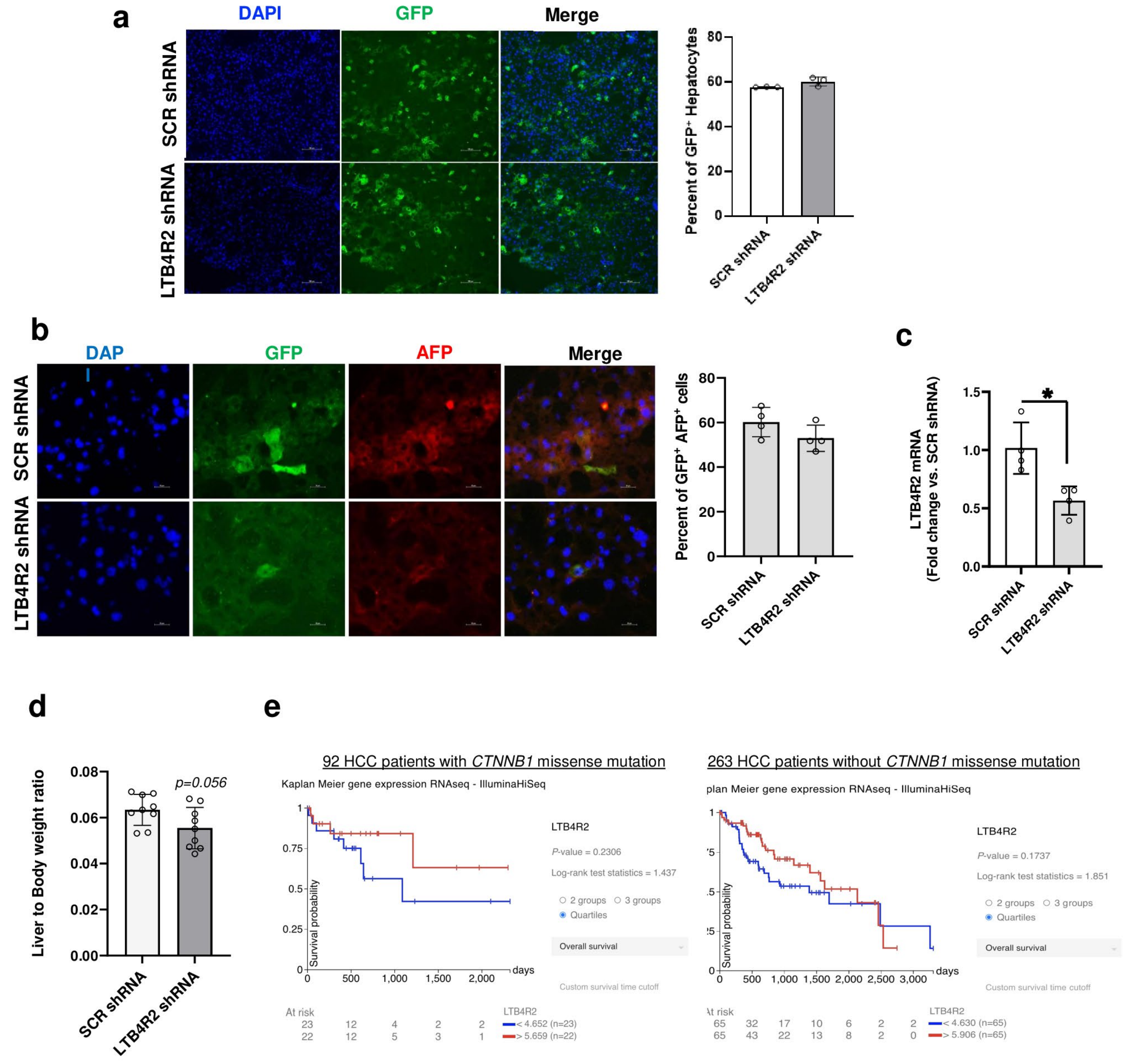


**Supplementary Figure 4. AAV8-based LTB4R2 targeting in mice and LTB4R2 translational relevance**. **a** IF microscopy images for GFP transduced by *SCR-shRNA* or *LTB4R2-shRNA* AAV8 vector in DEN+WAD mice and morphometric data for the percent of GFP+ hepatocytes to total hepatocytes. Data presented are means+SEM (n=3 liver sections). No statistical difference by two-sided t-test. **b** IF microscopy images of dual staining for GFP and AFP depicting GFP+AFP+ cells. A bar graph shows morphometric data for the percentage of GFP+AFP+ cells in the two groups. Data presented are means+SEM (n=4 liver sections). Two-sided t-test was performed. **c** *LTB4R2* mRNA in tumors of *SCR-shRNA* vs. *LTB4R2-shRNA* DEN+WAD mice. *p<0.05 vs. SCR-shRNA mice by two-sided t-test. Data presented are means+SEM (n=4 mouse samples each). An exact p value is provided in the Source data file. **d** Liver weight/body weight ratio of *SCR-shRNA* vs. *LTB4R2-shRNA* DEN+WAD mice. Data presented are means+SEM (n=9). Two-sided t-test was performed. **e** Kaplan-Meier survival curves of HCC patients with (left) or without (right) CTNNB1 missense mutation comparing between those with LTB4R2 high (red) vs. low (blue) expression the TCGA-LIHC (the Cancer Genome Atlas Liver Hepatocellular Carcinoma) cohort data. All relevant figures, source data and exact p values are provided in the Source Data file.
