# Supplementary material for: Hepatic stellate cell stearoyl co-A desaturase activates leukotriene B4 receptor 2 - β-catenin cascade to promote liver tumorigenesis
Source: Nat Commun. 2023 May 8;14:2651. doi: 10.1038/s41467-023-38406-8 (PMC10167314; doi:10.1038/s41467-023-38406-8)
Supplement: Supplementary file 3 — Description of Additional Supplementary Files [file 41467_2023_38406_MOESM3_ESM.pdf]

Description of Additional Supplementary Files

File Name: Supplementary Data 1
Description: RNA-seq data of Scd2f/f;CC vs Scd2f/f tumor adjacent liver (TAL).

File Name: Supplementary Data 2
Description: Summary of downregulated pathways of interest in Scd2f/f;CC vs. Scd2f/f TAL.

File Name: Supplementary Data 3
Description: scRNA-seq violin plot parameters (for Fig. 3c, 3f, 3g, Suppl. Fig 1b, 3h)

File Name: Supplementary Data 4
Description: PUFA metabolites lipidomic analysis raw data on Scd2f/f;CC vs. Scd2f/f (pmol/mg protein) (Fig. 1h).
