# Supplementary material for: Hepatic stellate cell stearoyl co-A desaturase activates leukotriene B4 receptor 2 - β-catenin cascade to promote liver tumorigenesis
Source: Nat Commun. 2023 May 8;14:2651. doi: 10.1038/s41467-023-38406-8 (PMC10167314; doi:10.1038/s41467-023-38406-8)
Supplement: Supplementary file 5 — Reporting Summary [file 41467_2023_38406_MOESM5_ESM.pdf]

nature portfolio

Corresponding author(s): Hidekazu Tsukamoto

Last updated by author(s): 2023 March 21, 2023

Reporting Summary

Nature Portfolio wishes to improve the reproducibility of the work that we publish. This form provides structure for consistency and transparency in reporting. For further information on Nature Portfolio policies, see our Editorial Policies and the Editorial Policy Checklist.

Statistics

For all statistical analyses, confirm that the following items are present in the figure legend, table legend, main text, or Methods section.

n/a	Confirmed	
☐	☒	The exact sample size (*n*) for each experimental group/condition, given as a discrete number and unit of measurement
☐	☒	A statement on whether measurements were taken from distinct samples or whether the same sample was measured repeatedly
☐	☒	The statistical test(s) used AND whether they are one- or two-sided<br>*Only common tests should be described solely by name; describe more complex techniques in the Methods section.*
☐	☒	A description of all covariates tested
☐	☒	A description of any assumptions or corrections, such as tests of normality and adjustment for multiple comparisons
☐	☒	A full description of the statistical parameters including central tendency (e.g. means) or other basic estimates (e.g. regression coefficient) AND variation (e.g. standard deviation) or associated estimates of uncertainty (e.g. confidence intervals)
☐	☒	For null hypothesis testing, the test statistic (e.g. *F*, *t*, *r*) with confidence intervals, effect sizes, degrees of freedom and *P* value noted<br>*Give P values as exact values whenever suitable.*
☒	☐	For Bayesian analysis, information on the choice of priors and Markov chain Monte Carlo settings
☒	☐	For hierarchical and complex designs, identification of the appropriate level for tests and full reporting of outcomes
☒	☐	Estimates of effect sizes (e.g. Cohen's *d*, Pearson's *r*), indicating how they were calculated

Our web collection on statistics for biologists contains articles on many of the points above.

Software and code

Policy information about availability of computer code

Data collection	FACS data were collected via FlowJo 10.8.1 version. RNA-seq data were analyzed by the Partek Flow software and Gencode M3 waas used to quantify the aligned reads to genes using Partek E/M method. For scRN-seq, raw sequencing data were converted to fastq files and de-multiplexed using the 10x Genomics' Cell Ranger software version 3.1.0.
Data analysis	scRNA-seq data analysis was performed with 10X Genomics' Loupe Browser sofware. For qPCR data analysis, Applied Biosystems ViATM 7 software, QuantStudio Software V1.3, was used. Confocal 3D images were reconstructed with IMARIS software (Bitplane, Zurich, Switzerland) while other IF images were captured by using Aperio ImageScope software. Kaplan Meier survival plots were generated by using the UCSC Xena browser and TCGA Illumina HiSeq data. Statistical analysis for differences between two sets of data was analyzed by two-tailed t-test by the Excel program and that for three or more groups by one way ANOVA with post-hoc Tukey test by using the Prism (GraphPad) or an on-line software (https://www.socscistatistics.com/tests/anova/default2.aspx).

For manuscripts utilizing custom algorithms or software that are central to the research but not yet described in published literature, software must be made available to editors and reviewers. We strongly encourage code deposition in a community repository (e.g. GitHub). See the Nature Portfolio guidelines for submitting code & software for further information.

Data

Policy information about availability of data

All manuscripts must include a data availability statement. This statement should provide the following information, where applicable:

- Accession codes, unique identifiers, or web links for publicly available datasets
- A description of any restrictions on data availability
- For clinical datasets or third party data, please ensure that the statement adheres to our policy

The RNA-seq and scRNA-seq data were deposited to the Gene Expression Omnibus with the accession code of GSE193980 (https://www.ncbi.nlm.nih.gov/geo/query/acc.cgi?acc=GSE193980) and GSE230843 (https://urldefense.com/v3/__https://www.ncbi.nlm.nih.gov/geo/query/acc.cgi?acc=GSE230843__;!!LIr3w8kk_Xxm!uA6VeDFs5BIGSTvbq90D3BgVv8FnUA7TMSbO1ggKJd8k4kmI4ankhsVdb-ySYpyTMuMEoghJpHHNwshdSg$).

Human research participants

Policy information about studies involving human research participants and Sex and Gender in Research.

Reporting on sex and gender	We used male and female HCC tissues available from the archival repositories as summarized in Supplementary Methods.
Population characteristics	The HCC tissues we used are from 55-81 years old males and females with th ethnicity of Hispanic and non-Hispanic White, Asian and the etiology background of viral hepatitis, alcoholic liver disease, and non-alcoholic steatohepatitis as summarized in Supplementary Methods.
Recruitment	No recruitment effort was made as these tissues are from archival tissue repositories.
Ethics oversight	Male patient HCC archival tissues and normal male liver tissues were obtained under the approved IRB protocols of University of Southern California (HS-16-00392), Kansas University Medical Center (11378), and Johns Hopkins University (00107893) for qPCR analysis and IHC analysis of HCC sections from male patients under the McGill University IRB (11-066 SDR). Male patient HCC organoids were established under the IRB protocol of Mt Sinai School of Medicine (20-04150).

Note that full information on the approval of the study protocol must also be provided in the manuscript.

Field-specific reporting

Please select the one below that is the best fit for your research. If you are not sure, read the appropriate sections before making your selection.

[x] Life sciences [ ] Behavioural & social sciences [ ] Ecological, evolutionary & environmental sciences

For a reference copy of the document with all sections, see nature.com/documents/nr-reporting-summary-flat.pdf

Life sciences study design

All studies must disclose on these points even when the disclosure is negative.

Sample size	For most in vitro or biochemical analyses, no prior sample size estimate was made. For in vivo studies, a sample size estimate was made by using the power analysis with an anticipated effect size, anticipated standard deviation, p=0.05 and the power of 80%.
Data exclusions	No data were excluded from experiments conducted and concluded with no procedural or technical flaws.
Replication	For culture studies and the sufficient number of each experiment was repeated to make sure the reproducibility and statistical conclusion of the effect. For mouse studies, the sufficient number of mice were used for analysis.
Randomization	Randamization of mice was performed for selection for different treat groups among males generated from the same litters. For testing two genotypes, mice born in the same litters were also used to maximally control other genetic variants.
Blinding	We analyzed samples as blindly as possible by assigning the serial sample numbers without the treatment/grouping information.

Reporting for specific materials, systems and methods

We require information from authors about some types of materials, experimental systems and methods used in many studies. Here, indicate whether each material, system or method listed is relevant to your study. If you are not sure if a list item applies to your research, read the appropriate section before selecting a response.

Materials & experimental systems

n/a	Involved in the study	
☐	☒	Antibodies
☐	☒	Eukaryotic cell lines
☒	☐	Palaeontology and archaeology
☐	☒	Animals and other organisms
☐	☒	Clinical data
☒	☐	Dual use research of concern

Methods

n/a	Involved in the study	
☒	☐	ChIP-seq
☐	☒	Flow cytometry
☒	☐	MRI-based neuroimaging

Antibodies

Antibodies used	Antibodies used are described in Suppl Table 2 including the source, catalog number, and dilutions used.
Validation	In addition to assessing the validation provided by the vendors fro each antibody, we pre-screened the quality of antibodies by performing immunoblotting and immunostaining with positive and negative control samples we generated.

Eukaryotic cell lines

Policy information about cell lines and Sex and Gender in Research

Cell line source(s)	Huh7 cells from Millipore Sigma (cat#01042712) and and LX2 cells from Fisher Scientific (cat# SCC064)
Authentication	Huh7 cells were authenticated by STR analysis but LX2 cells were not.
Mycoplasma contamination	Tested negative as required by the university biosafety guidelines.
Commonly misidentified lines (See ICLAC register)	No commonly misidentified lines used.

Animals and other research organisms

Policy information about studies involving animals; ARRIVE guidelines recommended for reporting animal research, and Sex and Gender in Research

Laboratory animals	Mouse: C57Bl/6j, Scd2f/f, Scd2f/f;Col1a1Cre; mTmG;Col1a1Cre on C57Bl/6i background. All 8 wk-32 wk old and housed as per approved housing conditions by Institutional Animal Care and Use Committee of the University of Southern California, including humidity range from 30 to 70%, temperature range from 20oC to 26oC and day/light cycle on time 6 am off time 6 pm.
Wild animals	No wild animals used.
Reporting on sex	We only used males as this sex has higher propensity to develop HCC and has previously used by us for Scd2f/f vs. Scd2f/f;Col1a1Cre mice for the liver cancer model.
Field-collected samples	No filed-collected samples were used.
Ethics oversight	This study was conducted according to the National Institute of Health guidelines and all animal experimentations and procedures were approved by the Institutional Animal Care and Use Committee of the University of Southern California (# 20426).

Note that full information on the approval of the study protocol must also be provided in the manuscript.

Clinical data

Policy information about clinical studies

All manuscripts should comply with the ICMJEguidelines for publication of clinical research and a completedCONSORT checklist must be included with all submissions.

Clinical trial registration	No clinical trial
Study protocol	*Note where the full trial protocol can be accessed OR if not available, explain why.*
Data collection	*Describe the settings and locales of data collection, noting the time periods of recruitment and data collection.*
Outcomes	*Describe how you pre-defined primary and secondary outcome measures and how you assessed these measures.*

Flow Cytometry

Plots

Confirm that:

- [x] The axis labels state the marker and fluorochrome used (e.g. CD4-FITC).
- [x] The axis scales are clearly visible. Include numbers along axes only for bottom left plot of group (a 'group' is an analysis of identical markers).
- [x] All plots are contour plots with outliers or pseudocolor plots.
- [x] A numerical value for number of cells or percentage (with statistics) is provided.

Methodology

Sample preparation	Liver cell isolation followed by gradient centrifugation for FACS separation
Instrument	Sorted on ARIA IIu (Beckton Dickson)
Software	FlowJo version 10.8.1 was used.
Cell population abundance	Cell number in different FACS fractions raining from 1,000 to 15,000.
Gating strategy	Solid state laser excited at 488nm and measured with 510/20BP emission mirrors for GFP and excited at 355nm and measured with 450/50BP mirrors for vitamin A

- [x] Tick this box to confirm that a figure exemplifying the gating strategy is provided in the Supplementary Information.
